# Supplementary figures and images for: Activation of actin-depolymerizing factor by CDPK16-mediated phosphorylation promotes actin turnover in Arabidopsis pollen tubes
Source: PLoS Biol. 2023 Apr 3;21(4):e3002073. doi: 10.1371/journal.pbio.3002073 (PMC10101649; doi:10.1371/journal.pbio.3002073)

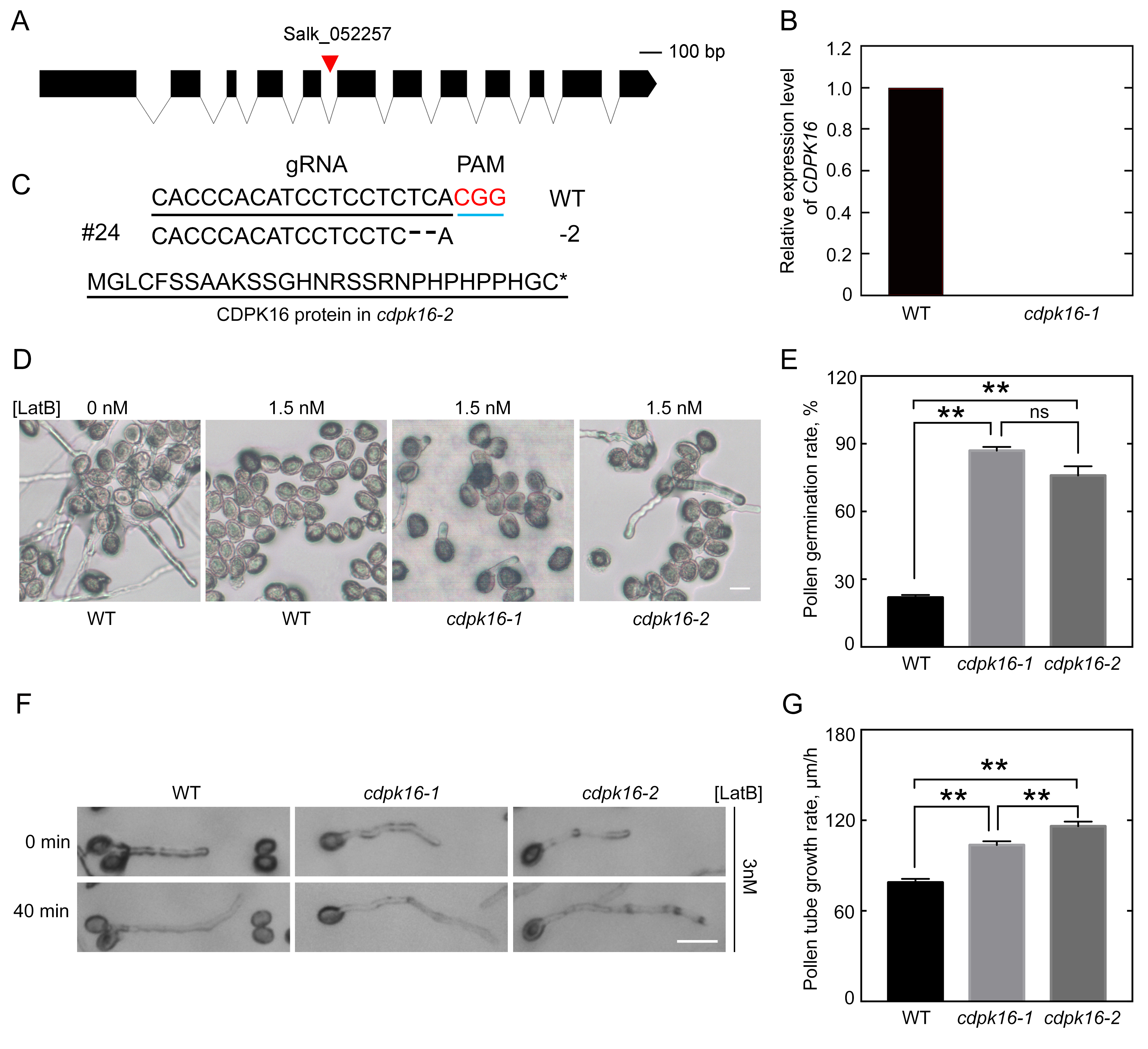

Supplement: S1 Fig — (A) Gene structure of CDPK16. CDPK16 contains 12 exons (black boxes) and 11 introns (black lines). The T-DNA insertion allele Salk_052257 was designated as cdpk16-1. The red triangle indicates the T-DNA insertion site. (B) Determination of the transcript level of CDPK16 in WT and cdpk16-1 by real-time quantitative RT-PCR. eIF4A was used as the internal control. Numerical data underlying this panel are available in S6 Data. (C) Creation of a CDPK16 knockout allele by the CRISPR/Cas9 approach. The mutant allele was designated as cdpk16-2. Deletion of 2 bases, T and C, in the first exon of the CDPK16 gene leads to early termination of protein translation (indicated by the asterisk). (D) Images of pollen grains and pollen tubes. Pollen derived from WT and cdpk16 mutants were germinated on GM in the presence or absence of 1.5 nM LatB. Bar = 25 μm. (E) Plot of pollen germination rate in the presence of LatB. Data are presented as mean ± SE, **P < 0.01 by Student’s t test. Numerical data underlying this panel are available in S6 Data. (F) Images of pollen tube growth at 2 time points. Single pollen tubes from WT and cdpk16 mutants in the presence of 3 nM LatB were selected for measurement. Bar = 20 μm. (G) Quantification of pollen tube growth rate from (F) in the presence of LatB. Data are presented as mean ± SE, **P < 0.01 by Student’s t test. Numerical data underlying this panel are available in S6 Data. (TIF) [file pbio.3002073.s001.tif]

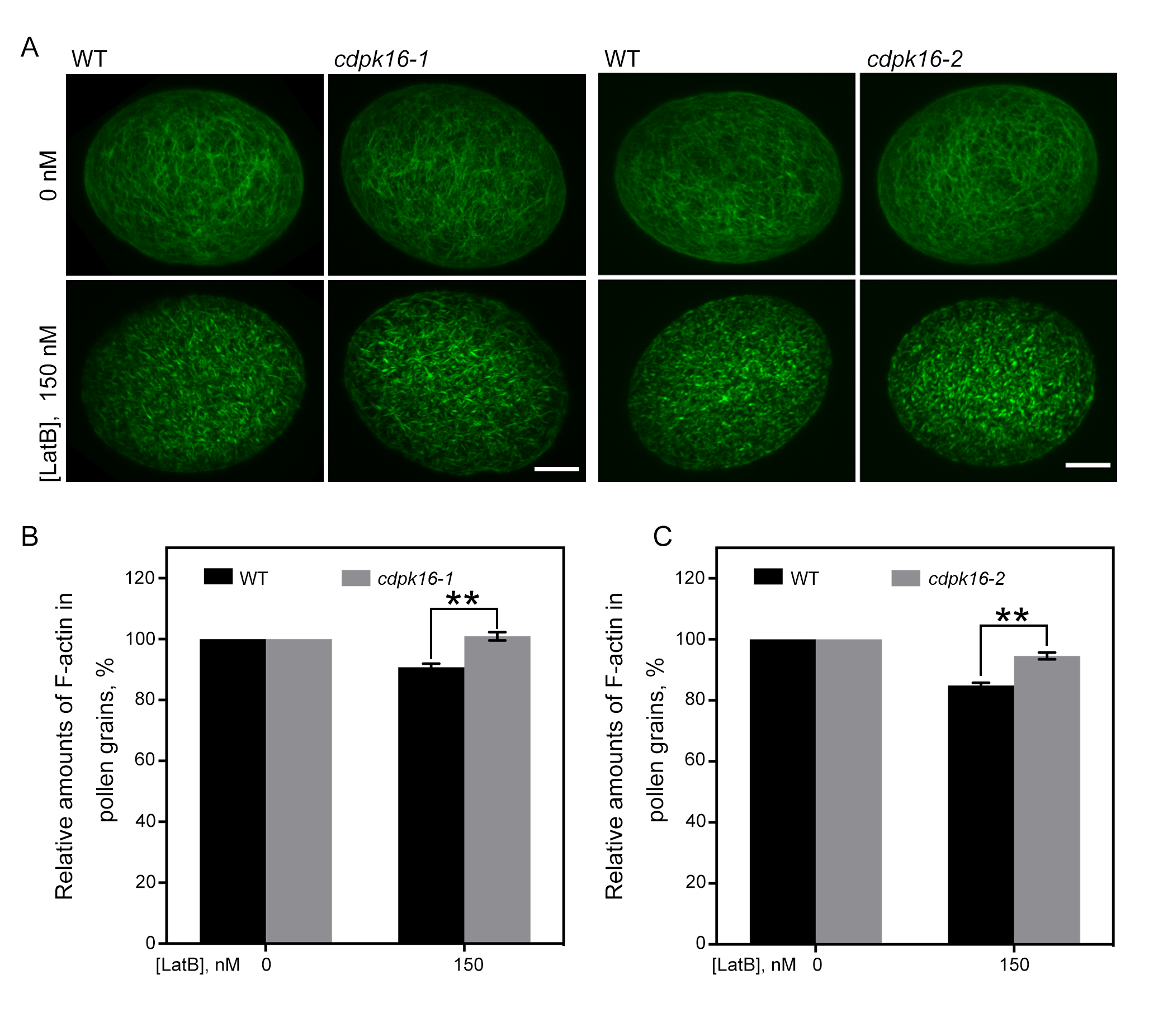

Supplement: S2 Fig — (A) Micrographs of the actin cytoskeleton in pollen grains. Pollen grains of WT and cdpk16 mutants were subjected to staining with Alexa-488 phalloidin in the presence or absence of 150 nM LatB. Bars = 5 μm. (B, C) Plots of the relative amount of F-actin in pollen grains. The amount of actin filaments was determined by measuring the fluorescence intensity of Alexa-488 phalloidin. The fluorescence intensity of Alexa-488 phalloidin in the absence of LatB was normalized to 100%. The data are presented as mean ± SE (n = 3). **P < 0.01 by Student’s t test. Numerical data underlying this panel are available in S7 Data. (TIF) [file pbio.3002073.s002.tif]

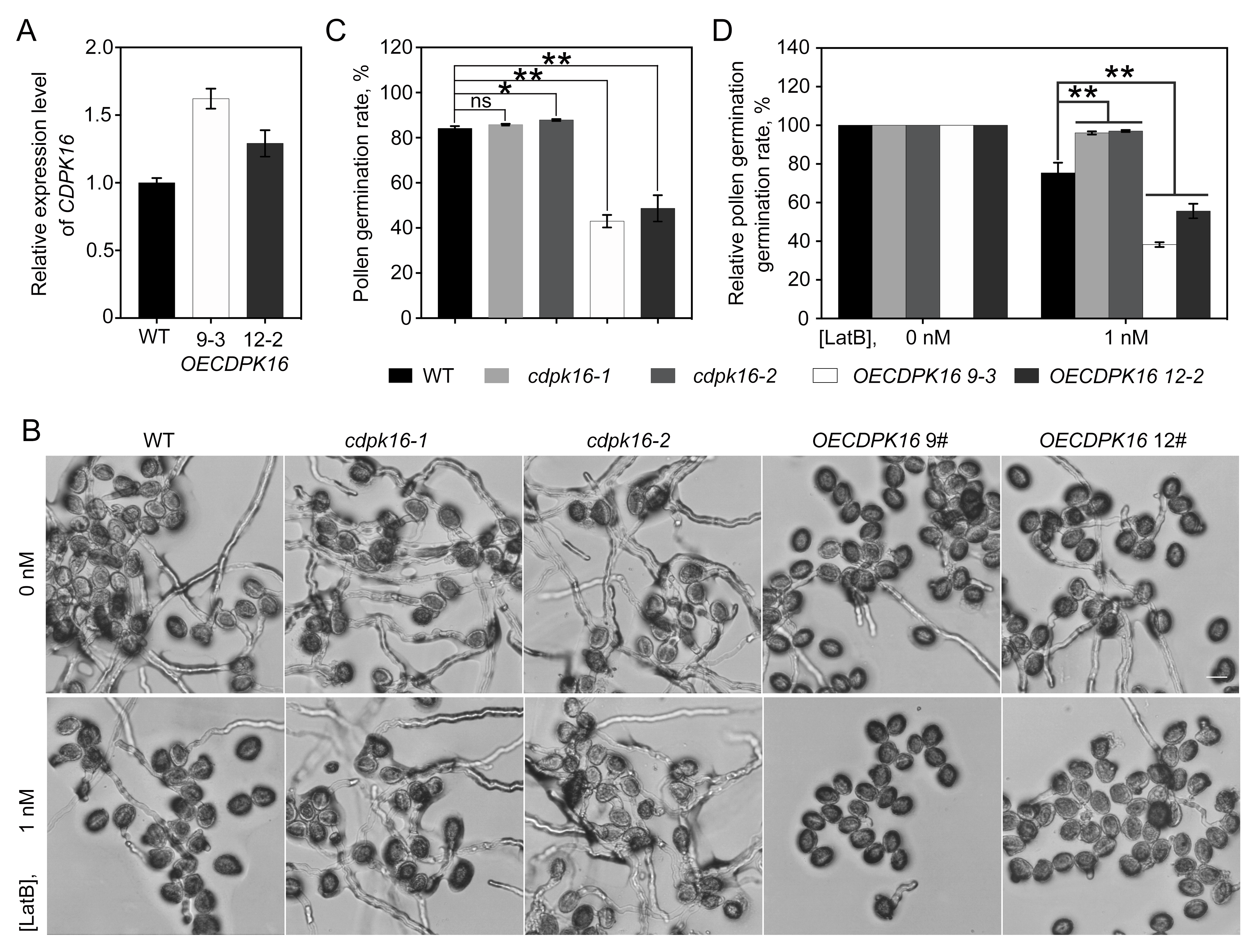

Supplement: S3 Fig — (A) Creation of CDPK16 overexpressors. The level of CDPK16 transcripts was determined by qRT-PCR analysis, and the amount of CDPK16 transcripts in WT was normalized to 1.0. Data are presented as mean ± SE, n = 3. Numerical data underlying this panel are available in S8 Data. (B) Micrographs of pollen grains and pollen tubes. Pollen derived from WT, cdpk16 mutants, and CDPK16 overexpressors were germinated on GM in the presence or absence of 1 nM LatB. Bar = 25 μm. (C) Quantification of pollen germination rates. Data are presented as mean ± SE, n = 3. ns, no significant difference, *P < 0.05, **P < 0.01 by Student’s t test. Numerical data underlying this panel are available in S8 Data. (D) Quantification of the relative pollen germination rate. Pollen germination rates in the absence of LatB were normalized to 100%. Data are presented as mean ± SE, n = 3. *P < 0.05, **P < 0.01 by ANOVA and Student’s t test. Numerical data underlying this panel are available in S8 Data. (TIF) [file pbio.3002073.s003.tif]

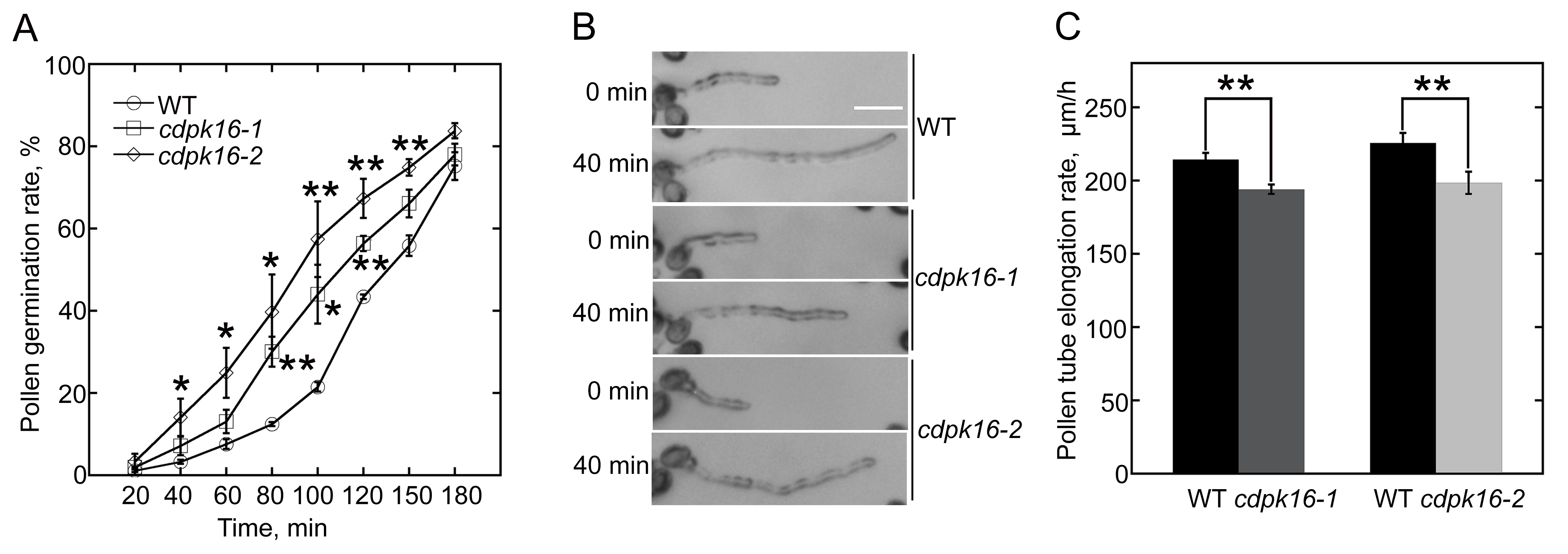

Supplement: S4 Fig — (A) Quantification of pollen germination rate at different time points. Data are presented as mean ± SE, *P < 0.05, **P < 0.01 by Student’s t test. Numerical data underlying this panel are available in S9 Data. (B) Images of pollen tube growth at 2 time points. Single pollen tubes from WT and cdpk16 mutants were selected for measurement. Bar = 20 μm. (C) Quantification of pollen tube growth rate from (B). Data are presented as mean ± SE, **P < 0.01 by Student’s t test. Numerical data underlying this panel are available in S9 Data. (TIF) [file pbio.3002073.s004.tif]

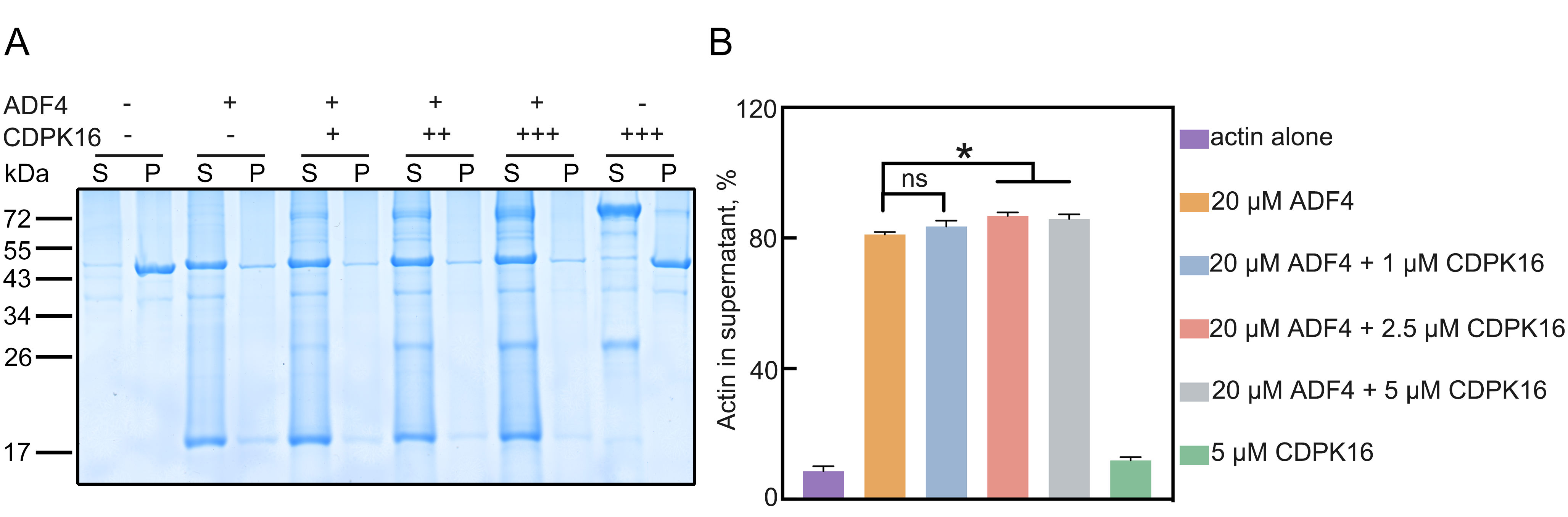

Supplement: S5 Fig — (A) SDS-PAGE analysis of the protein samples from a high-speed F-actin co-sedimentation experiment in the presence of Ca2+. F-actin, 3 μM; ADF4, 20 μM; CDPK16 (+), 1.0 μM; CDPK16 (++), 2.5 μM; CDPK16 (+++), 5.0 μM. The supernatant fractions (S) and pellets (P) were separated on SDS-PAGE gels, and proteins were detected by Coomassie Brilliant blue R 250 staining. The original pictures are available in S1 Raw Images. (B) Quantification of the amount of actin in the supernatant fractions shown in (A). Data are presented as mean ± SE, n = 3, *P < 0.05 and ns, no significant difference by Student’s t test. Numerical data underlying this panel are available in S10 Data. (TIF) [file pbio.3002073.s005.tif]

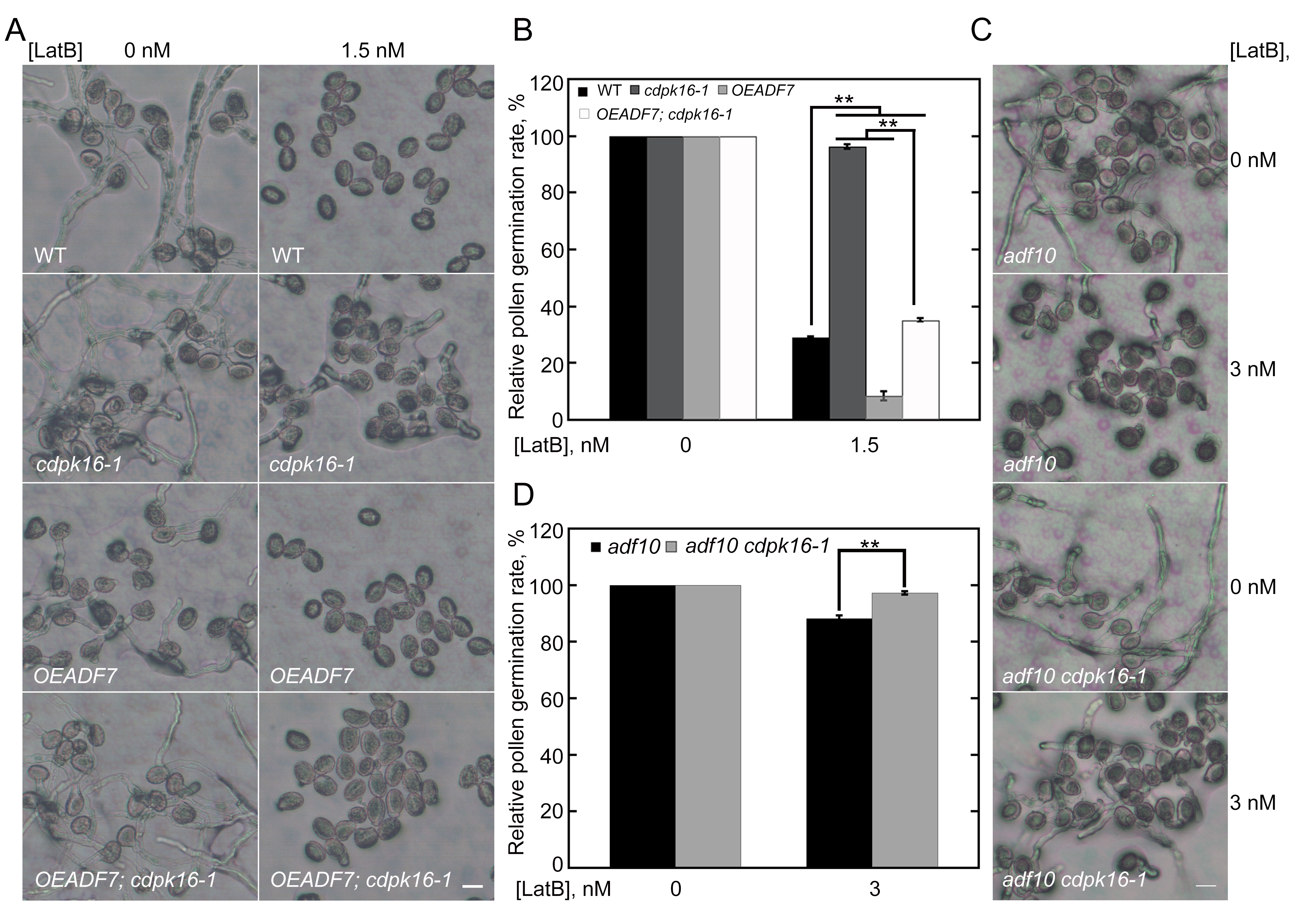

Supplement: S6 Fig — (A) Micrographs of pollen germinated on GM in the presence or absence of 1.5 nM LatB. Bar = 25 μm. (B) Overexpression of ADF7 alleviates the LatB-resistant pollen germination phenotype in cdpk16 mutants. The pollen germination rate of different genotypes in the absence of LatB was normalized to 100%. Data are presented as mean ± SE, n = 3. **P < 0.01 by Student’s t test. Numerical data underlying this panel are available in S11 Data. (C) Micrographs of pollen germinated on pollen germination medium in the presence or absence of 3 nM LatB. Bar = 25 μm. (D) Loss of function of CDPK16 enhances the LatB-resistant pollen germination phenotype in adf10 pollen. The pollen germination rate for different genotypes in the absence of LatB was normalized to 100%. Data are presented as mean ± SE, n = 3. **P < 0.01 by Student’s t test. Numerical data underlying this panel are available in S11 Data. (TIF) [file pbio.3002073.s006.tif]

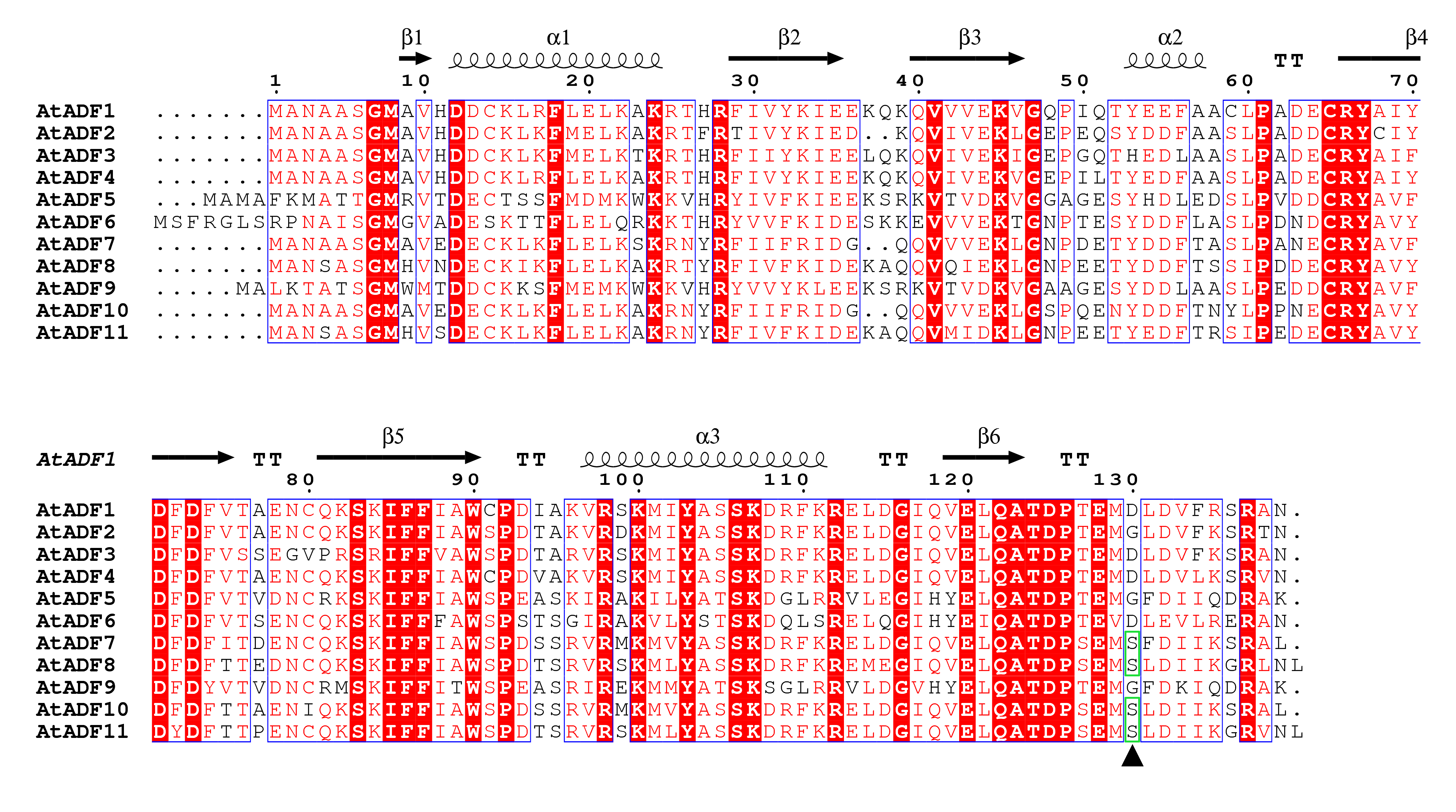

Supplement: S7 Fig — Protein sequence alignment of 11 Arabidopsis ADFs was performed using ESPript3. Green boxes and the black triangle indicate Ser128 in Arabidopsis class II ADFs. The predicted secondary structures are indicated above the sequence. The NCBI accession numbers for the sequences are as follows: Arabidopsis ADF1 (AtADF1), NP_190187; AtADF2, NP_566882; AtADF3, NP_851227; AtADF4, NP_851228; AtADF5, NP_565390; AtADF6, NP_565719; AtADF7, NP_194289; AtADF8, NP_567182; AtADF9, NP_195223; AtADF10, NP_568769; AtADF11, NP_171680. (TIF) [file pbio.3002073.s007.tif]

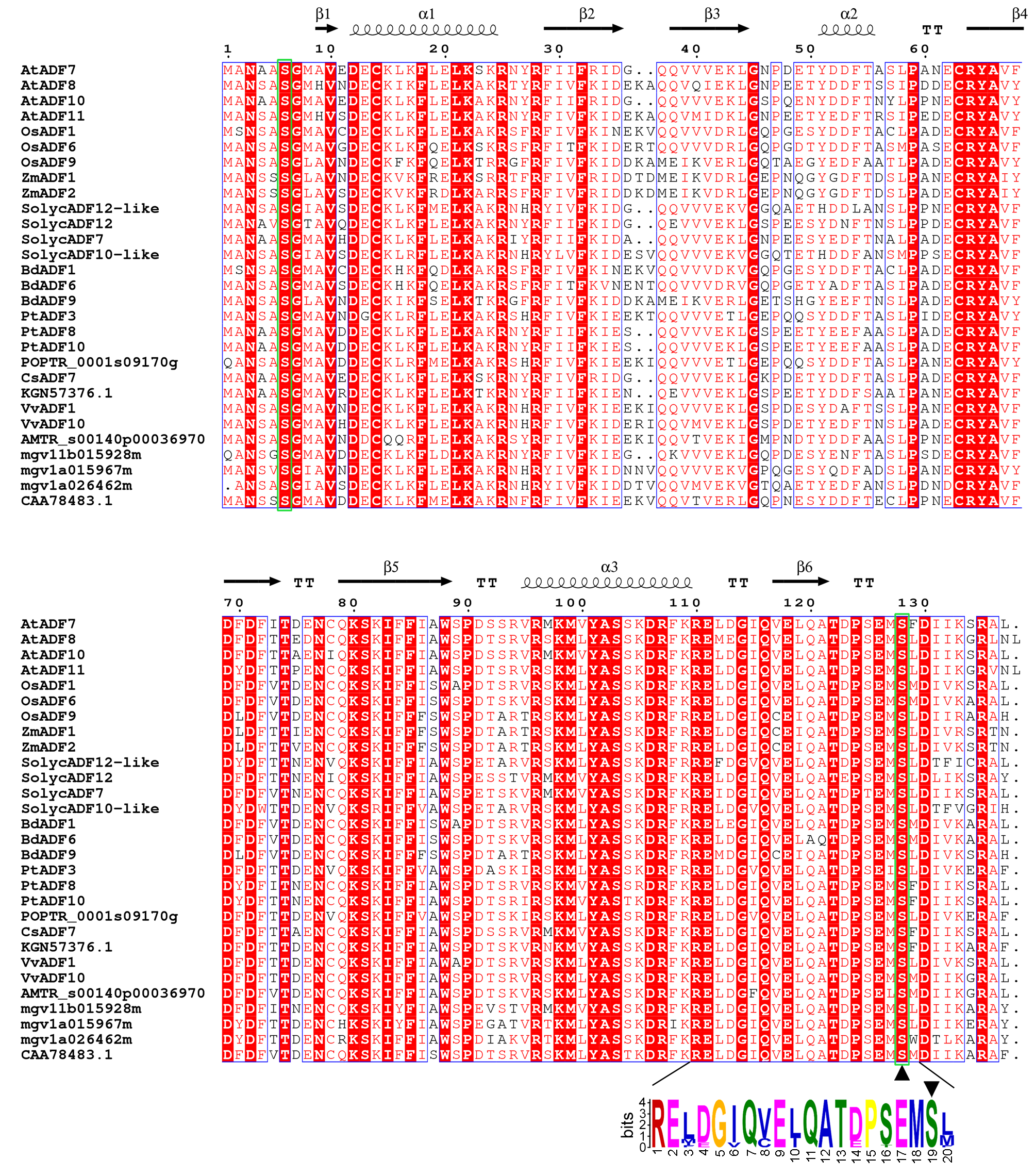

Supplement: S8 Fig — Protein sequence alignment of class II ADFs from Arabidopsis and other plant species was performed using ESPript3. Class II ADFs were selected according to previously published data [71–73]. The green boxes indicate 2 key conserved Serine residues (Ser6 and Ser128) that are associated with ADF function. Ser128 is also indicated by the black triangle. The peptide logo underneath the sequence alignment shows the amino acid prevalence at each position in the peptide sequence encompassing Ser128. The predicted secondary structures are indicated above the sequence. The GenBank accession numbers for ADFs are as follows: AtADF7, NP_194289; AtADF8, NP_567182; AtADF10, NP_568769; AtADF11, NP_171680; Oryza sativa ADF1 (OsADF1), Os02g0663800; OsADF6, Os04g0555700; OsADF9, Os07g0484200; Zea mays ADF1 (ZmADF1), ACG37280; ZmADF2, NP_001105590; Solanum lycopersicum ADF12 (SolycADF12), XP_010317604; SolycADF12-like, XP_015086534; SolycADF7, XP_004240732; SolycADF10-like, XP_019071428; Brachypodium distachyon ADF1 (BdADF1), XP_003570044; BdADF6, XP_014751334; BdADF9, XP_010238336; Populus trichocarpa ADF3 (PtADF3), XP_002303579; PtADF8, XP_002322471; PtADF10, XP_002318237; POPTR_0001s09170g, XP_002298043; Cucumis sativus ADF7 (CsADF7), XP_010448409; KGN57376.1, Csa_3G182120; Vitis vinifera ADF1 (VvADF1), XP_010662752; VvADF10, XP_002271495; AMTR_s00140p00036970, XP_006843087; mgv11b015928m; mgv1a015967m; mgv1a026462m; CAA78483.1. (TIF) [file pbio.3002073.s008.tif]

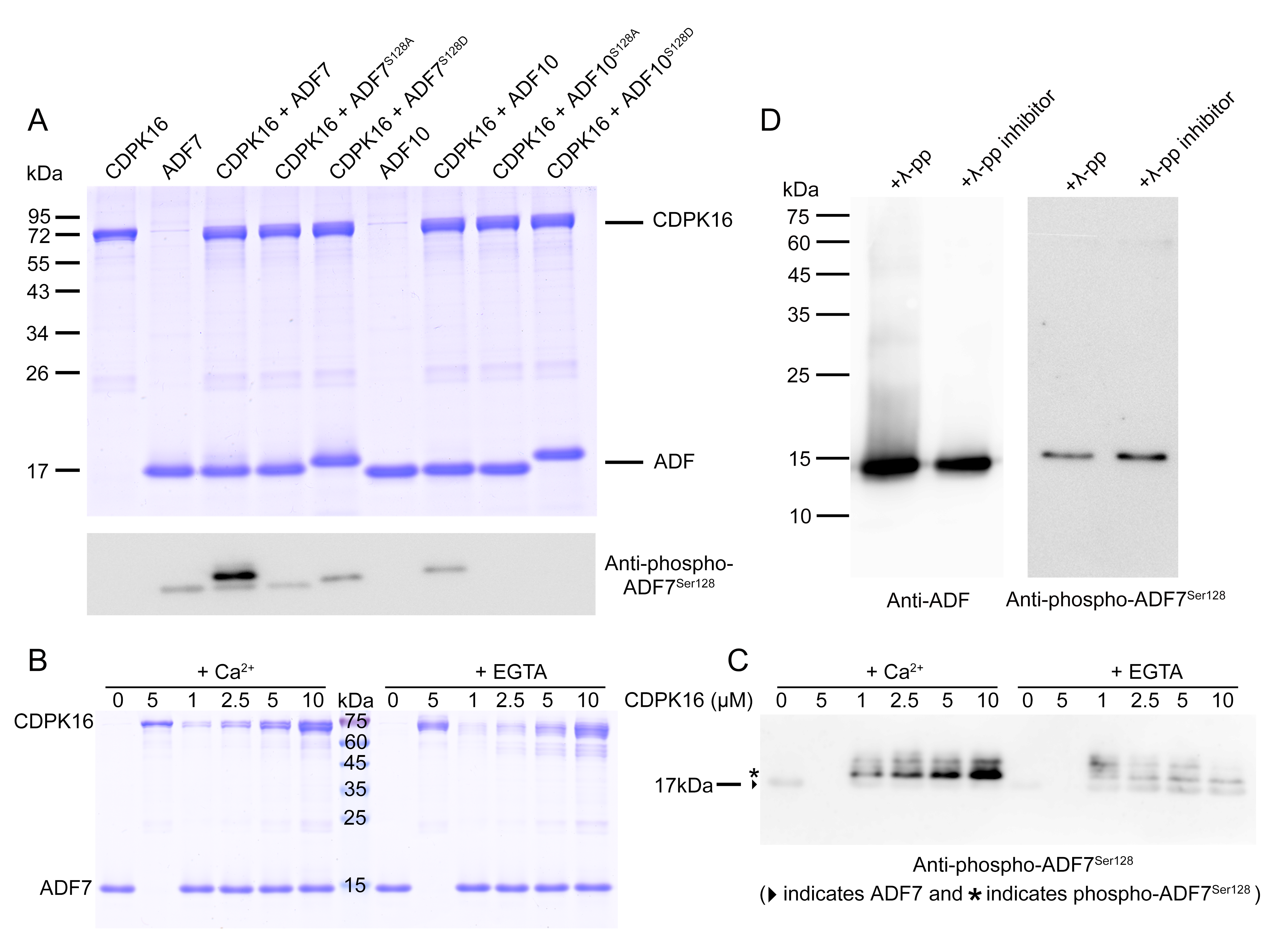

Supplement: S9 Fig — (A) The anti-phospho-ADF7(Ser128) antibody specifically recognizes CDPK16-phosphorylated ADF7. The original pictures are available in S1 Raw Images. (B, C) CDPK16 increases the amount of phosphorylated ADF7 in a dose- and calcium-dependent manner. (B) SDS-PAGE analysis of the proteins in the reaction. ADF7, 20 μM; CDPK16, 1.0 μM, 2.5 μM, 5.0 μM, 10 μM. (C) Western blot analysis of the protein samples shown in (B) probed with anti-phospho-ADF7(Ser128) antibody. The original pictures are available in S1 Raw Images. (D) Treatment with λpp (Lambda Protein Phosphatase) reduces the amount of phosphorylated ADF7 in pollen. Total proteins were isolated from mature pollen grains derived from proADF7::8His-gADF7; adf7. Western blot analysis of the pulled down 8His-ADF7 probed with anti-ADF7 antibody (left panel, as loading control) and anti-phospho-ADF7(Ser128) antibody (right panel). The original pictures are available in S1 Raw Images. (TIF) [file pbio.3002073.s009.tif]

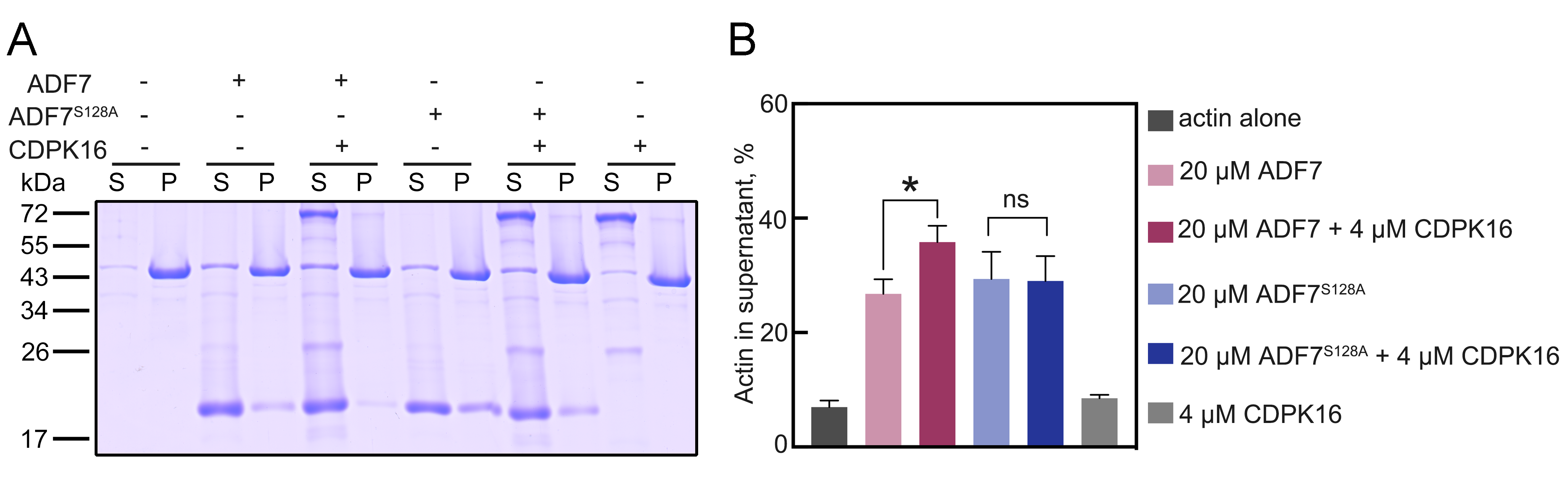

Supplement: S10 Fig — (A) SDS-PAGE analysis of the protein samples from a high-speed F-actin co-sedimentation experiment in the presence of Ca2+. F-actin, 3 μM; ADF7, 20 μM; CDPK16, 4.0 μM. The supernatant fractions (S) and pellets (P) were separated on SDS-PAGE gels, and proteins were detected by Coomassie Brilliant blue R 250 staining. The original pictures are available in S1 Raw Images. (B) Quantification of the amount of actin in the supernatant fractions. Data are presented as mean ± SE, n = 3, *P < 0.05 and ns, no significant difference by Student’s t test. Numerical data underlying this panel are available in S12 Data. (TIF) [file pbio.3002073.s010.tif]

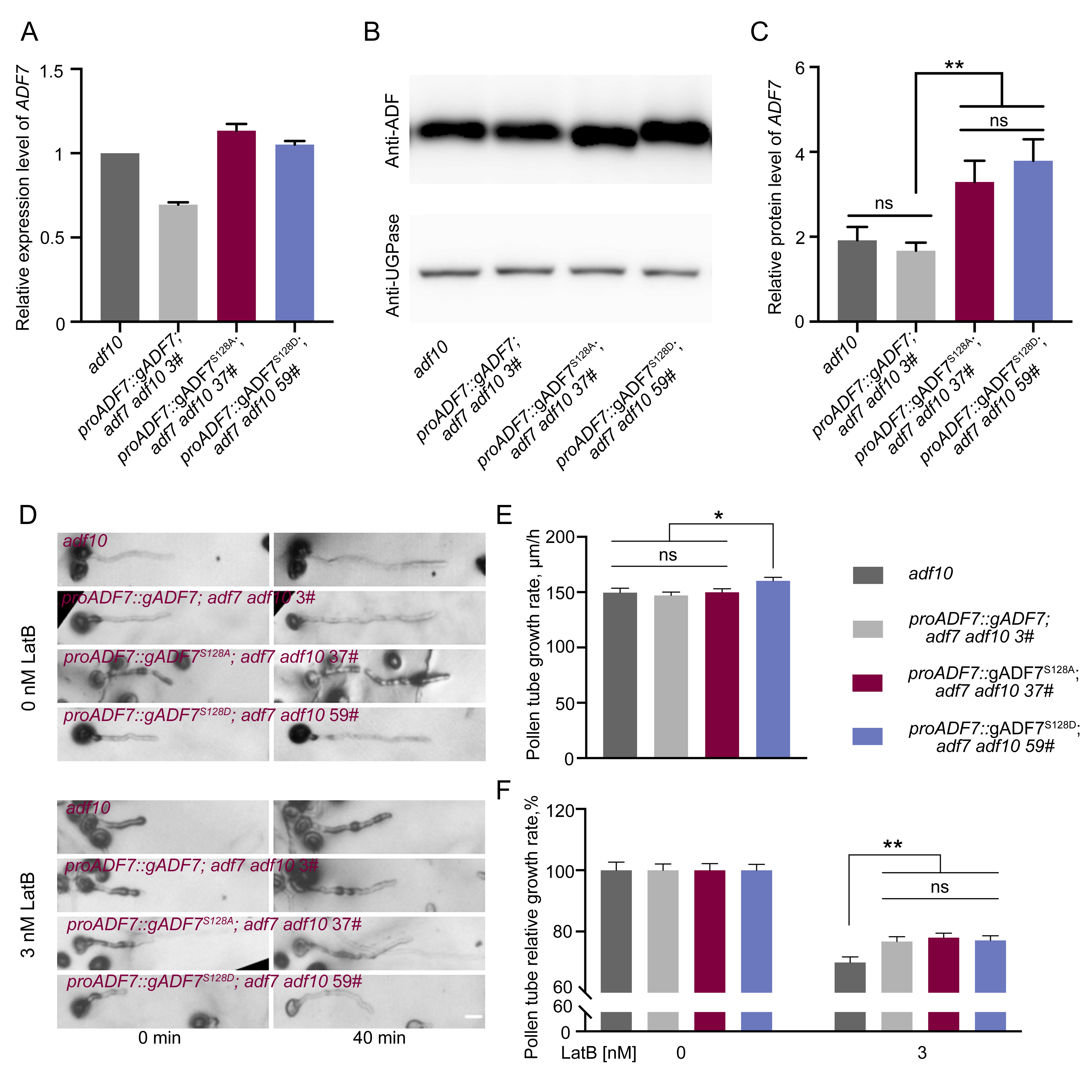

Supplement: S11 Fig — (A) qRT-PCR analysis to detect the amount of ADF7 transcripts in adf7 adf10 lines expressing WT ADF7, ADF7S128A, or ADF7S128D. Numerical data underlying this panel are available in S13 Data. (B) Western blot analysis to detect the amount of ADF7 in pollen. Blots were probed with antibodies against UGPase and ADF. The amount of UGPase was used to normalize the amount of ADF7, ADF7S128A, and ADF7S128D in pollen. The original pictures are available in S1 Raw Images. (C) Quantification of the amount of ADF7 protein in pollen. The relative amount of ADF7 protein from (B) is plotted. Data are presented as mean ± SE, n = 3, ns, no significant difference, **P < 0.01 by Student’s t test. Numerical data underlying this panel are available in S13 Data. (D) Micrographs of pollen tubes. Pollen tubes growing in the absence or presence of 3 nM LatB were presented. Individual pollen tubes at 2 different time points are shown. Bar = 25 μm. (E) Quantification of pollen tube growth rate in the absence of LatB. The growth rate of pollen tubes from (D, upper panels) is plotted. ns, no significant difference and *P < 0.05 by Student’s t test. Numerical data underlying this panel are available in S13 Data. (F) Quantification of relative pollen tube growth rate in the presence of 3 nM LatB. The growth rate of pollen tubes from (D, lower panels) is plotted. Data are presented as mean ± SE, n = 3, ns, no significant difference, **P < 0.01 by Student’s t test. Numerical data underlying this panel are available in S13 Data. (TIF) [file pbio.3002073.s011.tif]

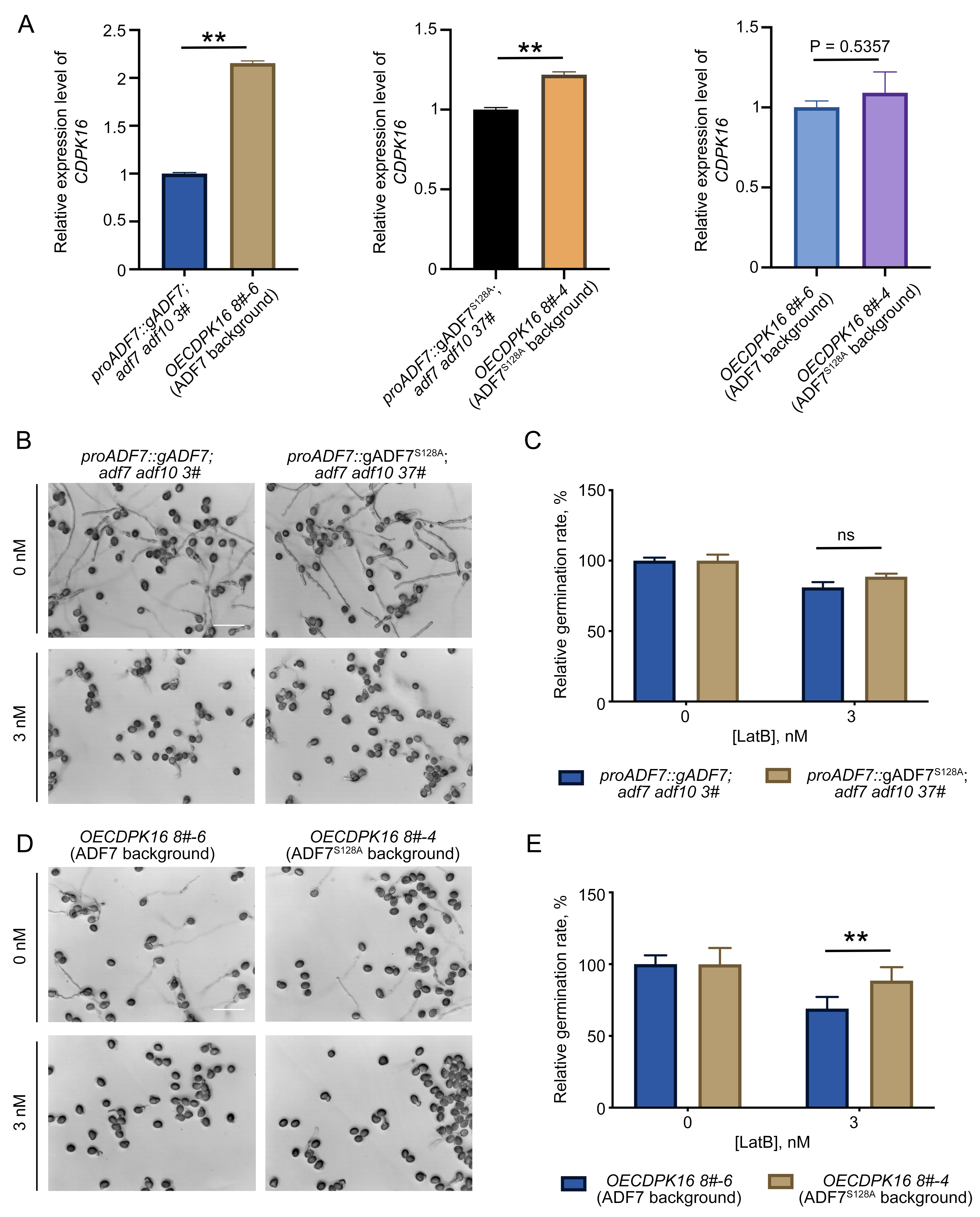

Supplement: S12 Fig — (A) qRT-PCR analysis to detect the amount of CDPK16 transcripts in adf7 adf10 lines expressing ADF7 or ADF7S128A. OECDPK16 8#-6 and OECDPK16 8#-4 are 2 CDPK16 overexpressors in the background of gADF7;adf7 adf10 and gADF7S128A;adf7 adf10, respectively, which have comparable amounts of CDPK16 transcripts. Numerical data underlying this panel are available in S14 Data. (B and D) Micrographs of pollen derived from gADF7;adf7 adf10, gADF7S128A;adf7 adf10 (B), OECDPK16 8#-6 and OECDPK16 8#-4 (D) in the absence and presence of 3 nM LatB. Scale bar = 100 μm. (C and E) Quantification of relative pollen germination rates. Data are the means of 3 replicates ± SE. ns, no significant difference, **P < 0.01 (Student’s t test). Numerical data underlying this panel are available in S14 Data. (TIF) [file pbio.3002073.s012.tif]

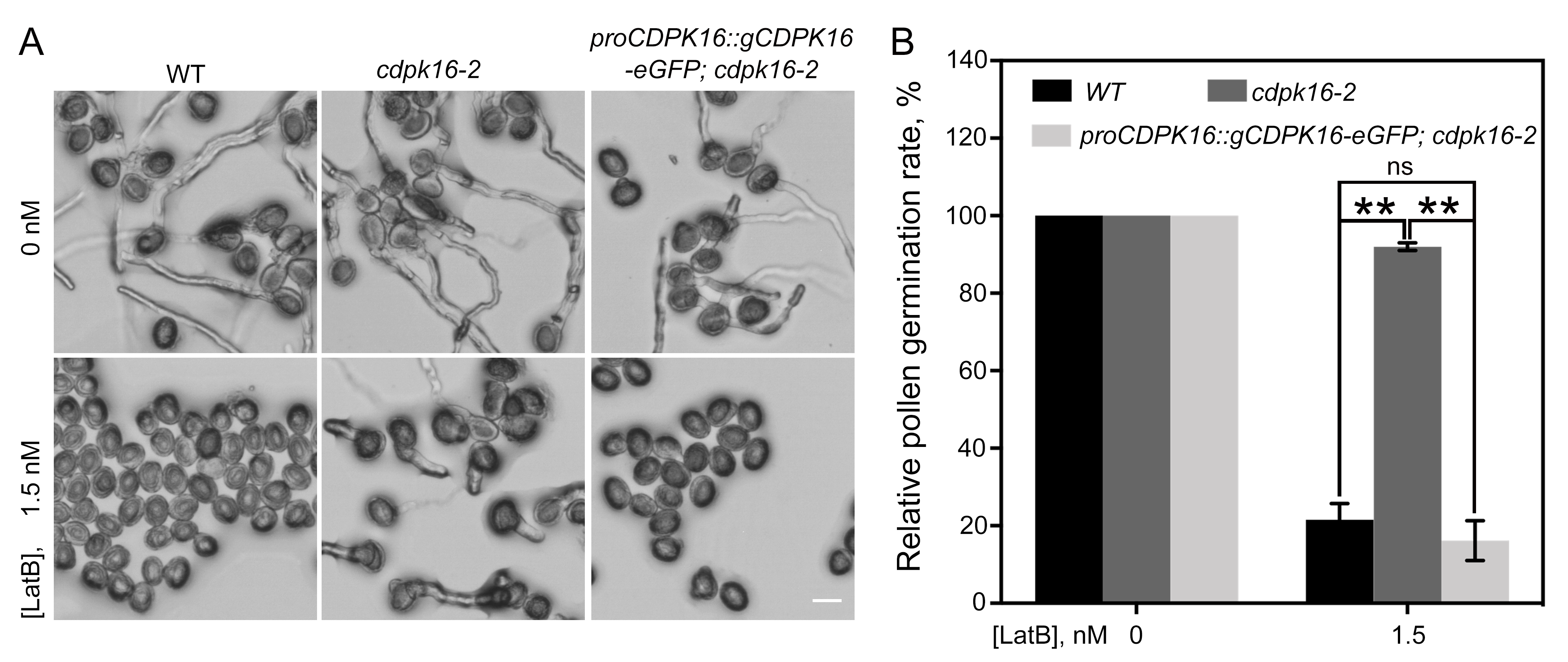

Supplement: S13 Fig — (A) Micrographs of pollen grains and pollen tubes. Pollen grains derived from WT, cdpk16-2, and the complementation line proCDPK16::gCDPK16-eGFP; cdpk16-2 were germinated on the surface of GM for 3 h in the presence or absence of 1.5 nM LatB. Bar = 25 μm. (B) Quantification of relative pollen germination rate. Pollen germination rate in the absence of LatB was normalized to 100%. Data are presented as mean ± SE, n ≥ 500, **P < 0.01 by Student’s t test. The experiments were repeated 3 times. Numerical data underlying this panel are available in S15 Data. (TIF) [file pbio.3002073.s013.tif]

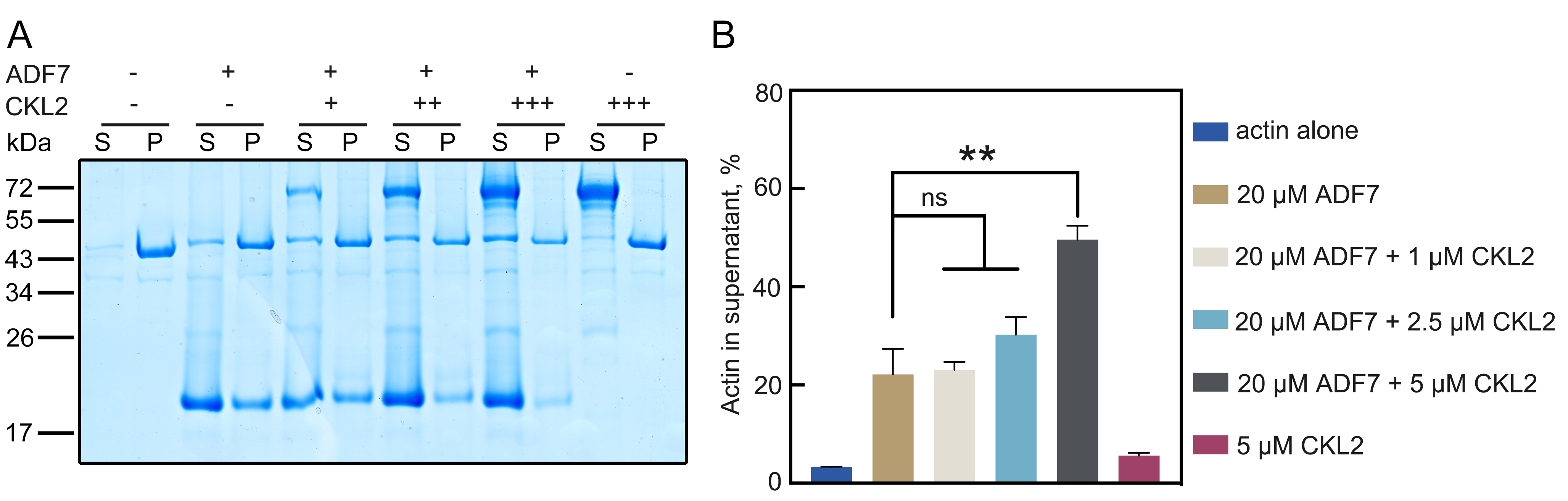

Supplement: S14 Fig — (A) SDS-PAGE analysis of the protein samples from a high-speed F-actin co-sedimentation experiment in the presence of Ca2+. F-actin, 3 μM; ADF7, 20 μM; CKL2 (+), 1.0 μM; CKL2 (++), 2.5 μM; CKL2 (+++), 5.0 μM. The supernatant fractions (S) and pellets (P) were separated on SDS-PAGE gels, and proteins were detected by Coomassie Brilliant blue R 250 staining. The original pictures are available in S1 Raw Images. (B) Quantification of the amount of actin in the supernatant fractions shown in (A). Data are presented as mean ± SE, n = 3, **P < 0.01 and ns, no significant difference by Student’s t test. Numerical data underlying this panel are available in S16 Data. (TIF) [file pbio.3002073.s014.tif]
