# Supplementary material for: Activation of actin-depolymerizing factor by CDPK16-mediated phosphorylation promotes actin turnover in Arabidopsis pollen tubes
Source: PLoS Biol. 2023 Apr 3;21(4):e3002073. doi: 10.1371/journal.pbio.3002073 (PMC10101649; doi:10.1371/journal.pbio.3002073)
Supplement: S1 Table — (DOCX) [file pbio.3002073.s015.docx]

**S1 Table. Primers used in this study.**

| Primer ID | Primer Sequences (5’→3’) |
| --- | --- |
| **PG1** | **CTGCAG**TATTGAATTTGTCTTTACAA |
| **PG2** | **GGTACC**GACCTTGCGAGAAATAAG |
| **E1** | GG**GGTACC**ATGGTGAGCAAGG |
| **E2** | G**GAATTC**TTACTTGTACAGCTCG |
| **O1** | CG**GGATCC**ATGGCGAACGCGGCGTCG |
| **O2** | AAC**GAATTC**TTAGAGAGCTCGGCTTTTG |
| **O3** | **GTCGAC**ATGGGTCTCTGTTTCTCCT |
| **O4** | **GAATTC**TTAGACCTTGCGAGAAATAAG |
| **O5** | GCTAAGCTCTAGTGATAGAAGCTTATACTCGACTCAGAAG |
| **O6** | GCCAAATGTTTGAACGGAATTCTTAGACCTTGCGAGAAATAAG |
| **CR1** | ATATATGGTCTCGATTGCACCCACATCCTCCTCTCAGTT |
| **CR2** | TGCACCCACATCCTCCTCTCAGTTTTAGAGCTAGAAATAGC |
| **CR3** | AACGTGCGGTGGCGTATGCCGCCAATCTCTTAGTCGACTCTAC |
| **CR4** | ATTATTGGTCTCGAAACGTGCGGTGGCGTATGCCGCCAA |
| **CR5** | ATGGGTCTCTGTTTCTCCTC |
| **CR6** | CTTGGCCTTATCGATCTTC |
| **PG3** | CG**GGATCC**ATGCATCACCATCACCATCACCATCACGTTTGTTCTCTTCTCTTTTTC |
| **PG4** | GG**GGTACC**TCCTTTCTAATGTGCGTTG |
| **M1** | GCCACTGATCCTAGCGAGATG**GCT**TTCGACATTATCAAAAGCCGA |
| **M2** | TCGGCTTTTGATAATGTCGAA**AGC**CATCTCGCTAGGATCAGTGGC |
| **M3** | GCCACTGATCCTAGCGAGATG**GAT**TTCGACATTATCAAAAGCCGA |
| **M4** | TCGGCTTTTGATAATGTCGAA**ATC**CATCTCGCTAGGATCAGTGGC |
| **M5** | ACTGACCCGAGCGAGATG**GCT**CTCGACATCATCAAAAG |
| **M6** | CTTTTGATGATGTCGAG**AGC**CATCTCGCTCGGGTCAGT |
| **M7** | CACTGACCCGAGCGAGATG**GAT**CTCGACATCATCAAAAGT |
| **M8** | ACTTTTGATGATGTCGAG**ATC**CATCTCGCTCGGGTCAGTG |
| **R1** | CGATGTGCAGCAAGTCTCTC |
| **R2** | CTCCCGAACCTTCCACTTCT |
| **R3** | AGCACTATCACATCCATGGG |
| **R4** | ATCTTTCGCTAGAGCCTGCC |
| **R5** | TATCATCGATCCTGATCTTCAC |
| **R6** | CTCCCAAACGGAAGTAGAAAGA |
| **LCI1** | GG**GGTACC**ATGGGTCTCTGTTTCTCC |
| **LCI2** | ACGC**GTCGAC**GACCTTGCGAGAAATAAG |
| **LCI3** | GG**GGTACC**ATGGCGAACGCGGCGTC |
| **LCI4** | ACGC**GTCGAC**CTAGAGAGCTCGGCTTTTG |
| **P1** | **GAATTC**ATGGCGAACGCGGCGTC |
| **P2** | **GTCGAC**GAGAGCTCGGCTTTTGATAATG |
| **P3** | **GAATTC**ATGGGTCTCTGTTTCTCCTC |
| **P4** | **GTCGAC**TTAGACCTTGCGAGAAATAA |

The black bold and underlined letters indicate restriction sites, and the red bold and underlined letters indicate point mutation sites.
