# Supplementary material for: Activation of actin-depolymerizing factor by CDPK16-mediated phosphorylation promotes actin turnover in Arabidopsis pollen tubes
Source: PLoS Biol. 2023 Apr 3;21(4):e3002073. doi: 10.1371/journal.pbio.3002073 (PMC10101649; doi:10.1371/journal.pbio.3002073)

Fig. 2E, SDS-PAGE and Autorad image

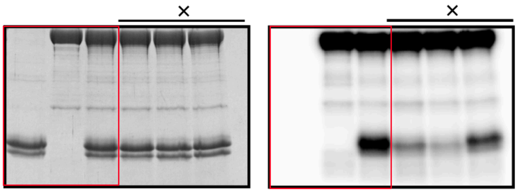

Fig.2F, 2-DE image

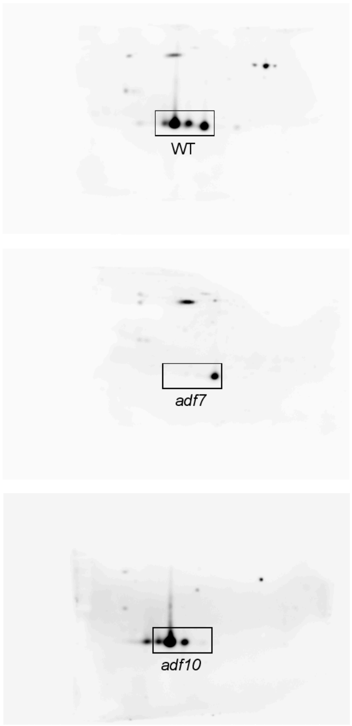

Fig.2G, 2-DE image

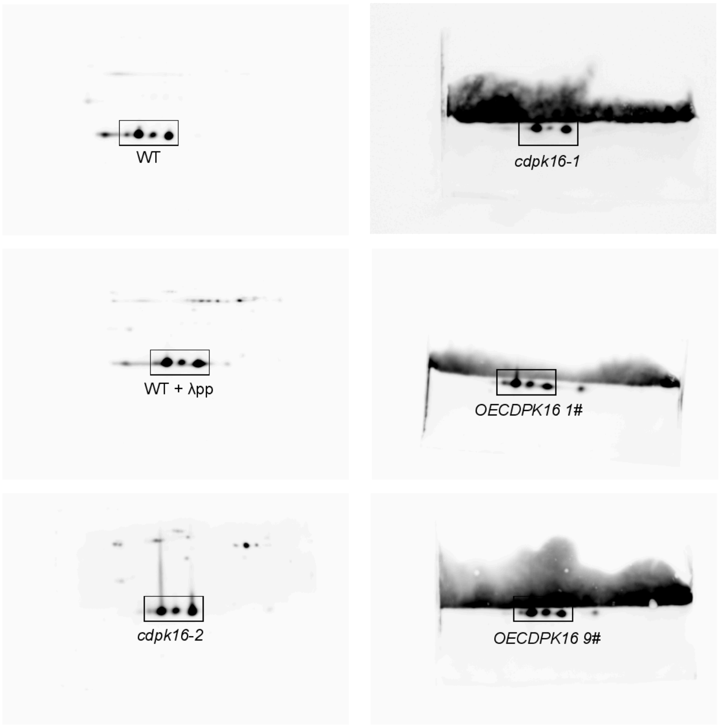

Fig.3A, SDS-PAGE image, cropped image shown inset.

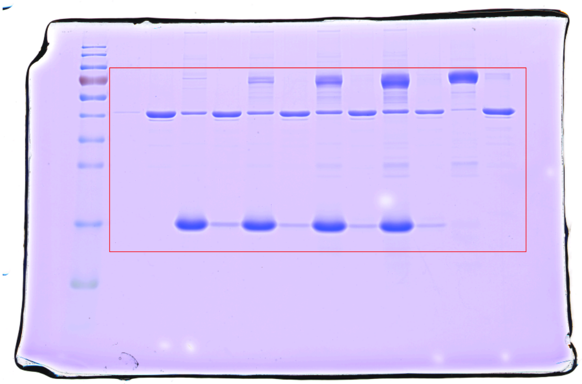

Fig.3C, SDS-PAGE image

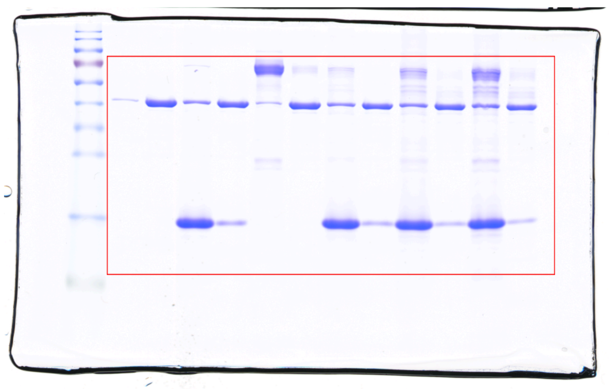

Fig.4A, SDS-PAGE image, cropped image shown inset.

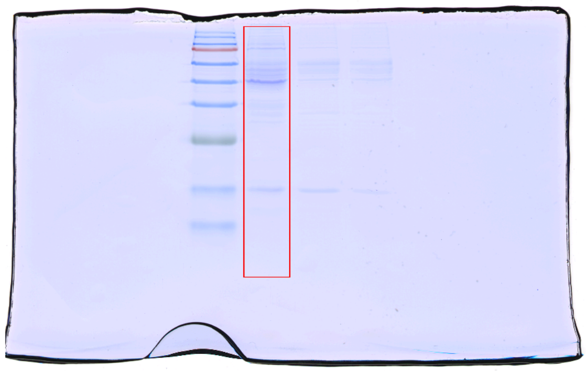

Fig.4B, SDS-PAGE image.

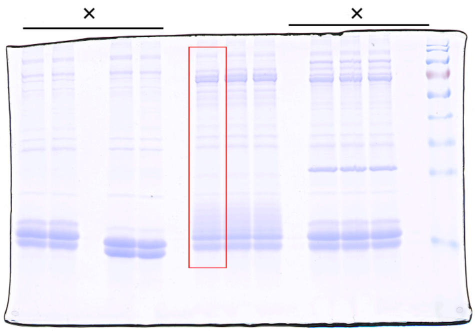

Fig. 4C, SDS-PAGE, cropped image shown inset.

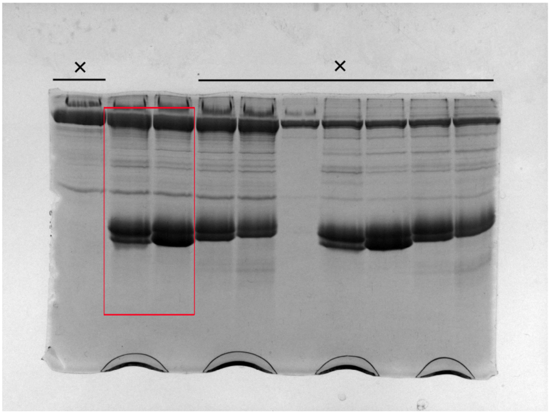

Fig. 4C, Autorad image

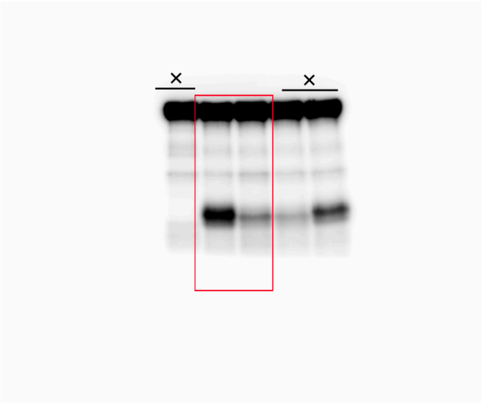

Fig.4E, 2-DE image, cropped image shown inset.

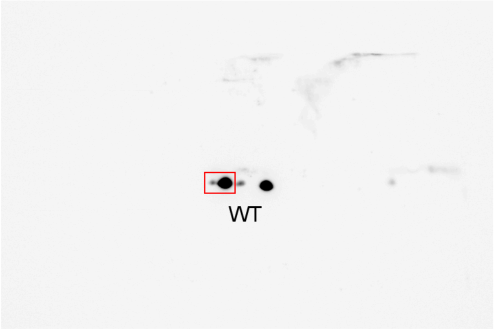

Fig.4E, 2-DE image

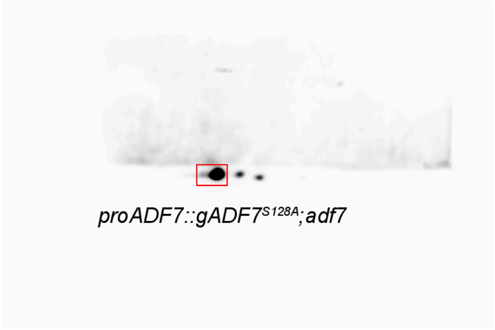

Fig.4E, 2-DE image

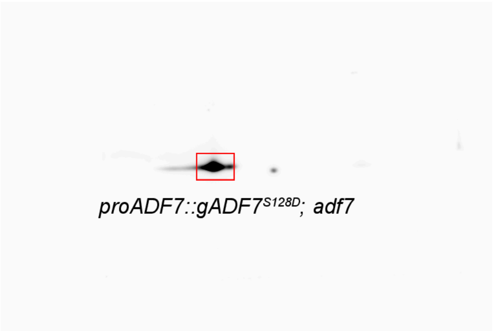

Fig.4F, SDS-PAGE image, cropped image shown inset.

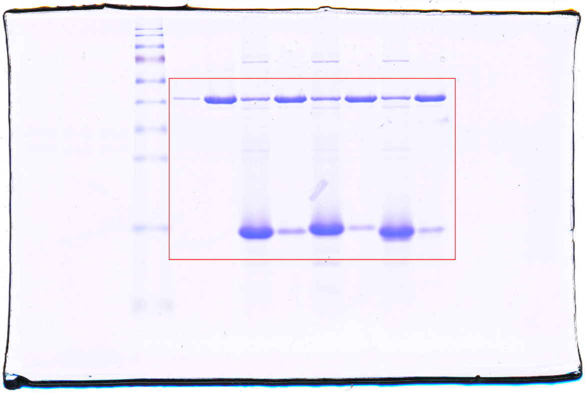

Fig. S5A, SDS-PAGE, cropped image shown inset.

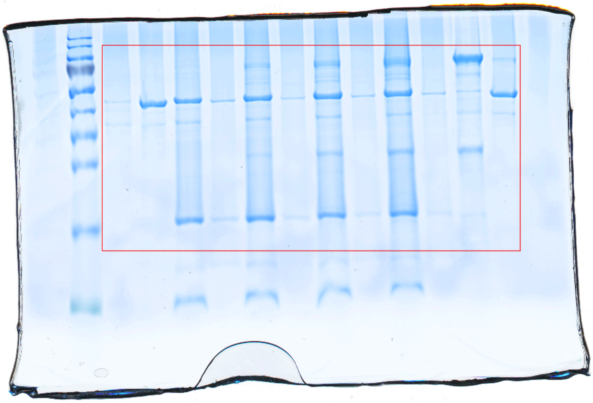

Fig.S9A, SDS-PAGE image.

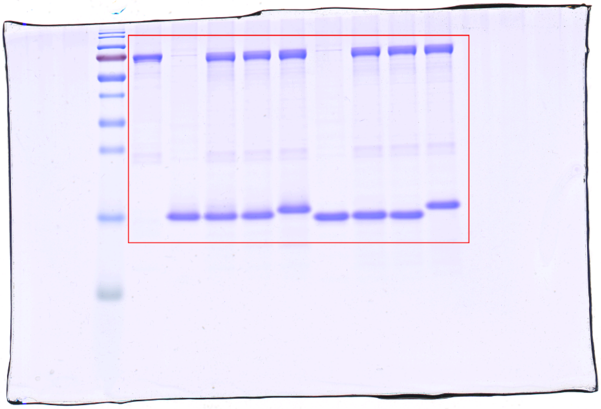

Fig.S9B, SDS-PAGE image.

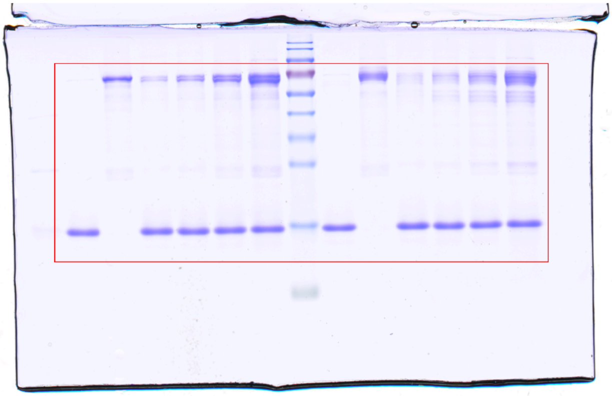

Fig.S9A, western blot image.

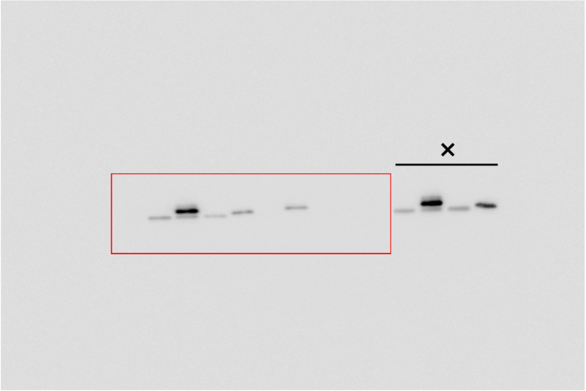

Fig.S9C, western blot image.

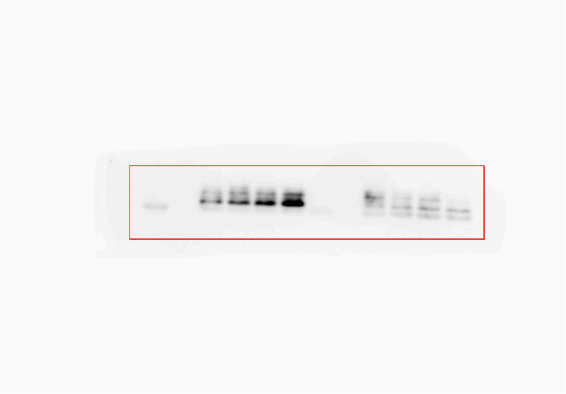

Fig.S9D, western blot image.

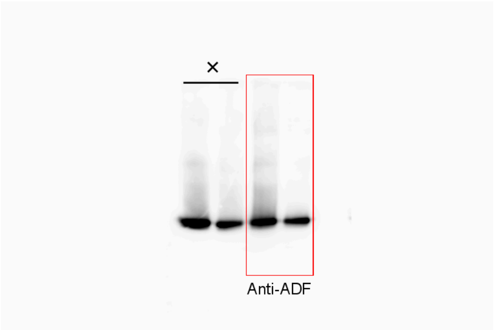

Fig.S9D, western blot image.

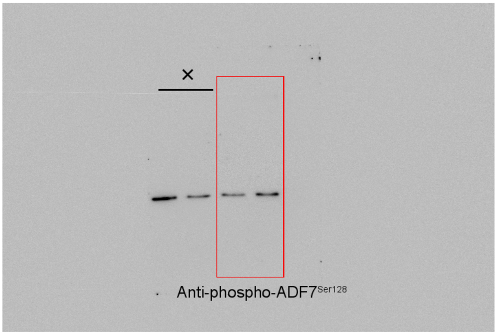

Fig. S10A, SDS-PAGE

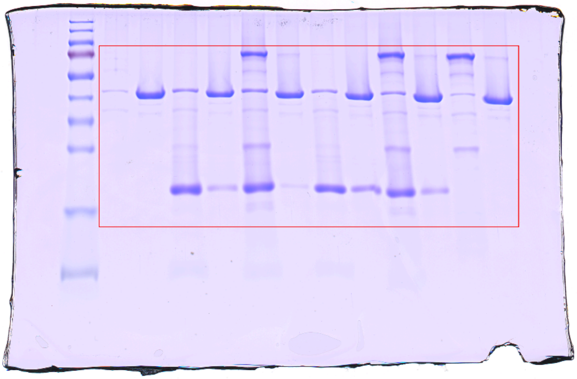

Fig. S11B, western-blot, cropped image shown inset.

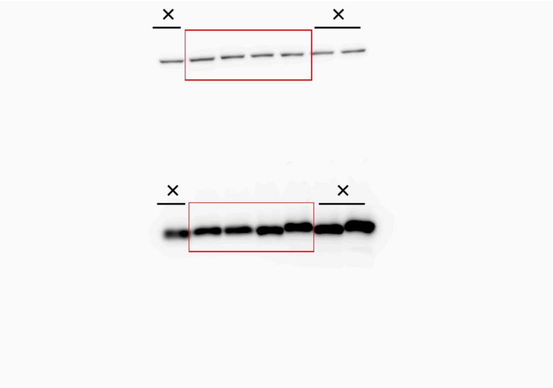

Fig. S14A, SDS-PAGE image.

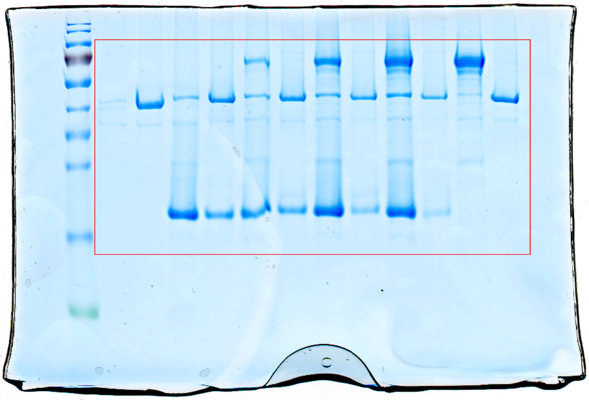

Supplement: S1 Raw Images — (PDF) [file pbio.3002073.s032.pdf]
