# Supplementary material for: Activation of actin-depolymerizing factor by CDPK16-mediated phosphorylation promotes actin turnover in Arabidopsis pollen tubes
Source: PLoS Biol. 2023 Apr 3;21(4):e3002073. doi: 10.1371/journal.pbio.3002073 (PMC10101649; doi:10.1371/journal.pbio.3002073)
Supplement: Attachment — Submitted filename: Response to reviewer commments.docx [file pbio.3002073.s043.docx]

**Response to Reviewers’ Comments**

We thank the three anonymous reviewers for their constructive comments and suggestions. We have taken the comments seriously and we have addressed the raised concerns and criticisms carefully. Details of the additions and changes can be found in the revised manuscript. With those revisions, we believe that the quality of our manuscript has been improved substantially. Below, we itemize our detailed responses to the comments and criticisms.

Reviewers' comments

Rev. 1:

*This manuscript entitled "Phosphorylation-mediated Activation of Actin-depolymerizing Factor in Arabidopsis" by Wang et al provided a molecular link between Ca2+/CDPK signaling and actin cytoskeleton organization in pollen tubes. Previous studies have found that plant ADFs are phosphorylated and inactivated by CDPK(s), whereas this paper reveals that CDPK16-mediated phosphorylation upregulates ADF7's activity in promoting actin depolymerizing and severing at the apical and subapical regions of pollen tubes.*

*Through providing substantial evidence both in vivo and in vitro, the authors enhance our understanding of the regulation of ADF in plant community. This paper is well-organized and falls within the scope of PLOS Biology.*

Response: Thanks for the encouraging comments.

*To further improve the manuscript, I have listed some remarks.*

*1. The authors said that they performed a forward chemical genetic screening for mutants showing alteration in LatB sensitivity and found one mutant allele of CDPK16. More details should be added for how the gene being identified.*

Response: To carry out the screen, we ordered the pool of homozygous T-DNA insertion alleles from NASC, and selected individual T-DNA insertion alleles of genes that showed a reasonable level of expression in pollen guided by the gene expression data. We then performed the pollen germination experiments to screen for mutants with a LatB-resistant pollen germination phenotype. We added this information about the screening procedure in the Materials and Methods section. Please see the revised manuscript for the newly added information.

*2. The subhead "Overexpression of ADF7 Alleviates the Actin Turnover Defects in cdpk16 Pollen, and Loss of Function of CDPK16 Enhances the Actin Turnover Defects in adf10 Pollen". The phenotype they provided in Fig S5 is only about pollen germination, so the word "Actin Turnover Defects" is not accurate. This also exist in other parts of the manuscript (e.g. Fig S9), please confirm and modify them*.

Response: Thanks for pointing this out. We fixed the problems throughout the manuscript.

*3. In Fig 5A, they showed that actin filaments were brighter in proADF7::gADF7S128A; adf7 adf10 pollen tubes than in adf10 and proADF7::gADF7; adf7 adf10 pollen tubes. However, the in vitro assays in Fig 4 found that ADF7S128A had roughly similar activity to ADF7. How to explain the difference between the function of ADF7S128A in vivo and in vitro? Moreover, I noticed that they have the plant material of proADF7::gADF7S128D; adf7 adf10. And the actin filament organization of this plant material also need to be displayed and quantified in Fig 5 to better support the in vitro evidence.*

Response: We believe that the actin depolymerizing activity of ADF7 can be upregulated by phosphorylation at its Ser128. The actin depolymerizing activity of ADF7^S128A^ fails to be upregulated by phosphorylation *in vivo*, although ADF7^S128A^ and ADF7 have roughly similar actin depolymerizing activity *in vitro*. This explains why actin filaments were brighter in *proADF7::gADF7S128A; adf7 adf10* pollen tubes than in *adf10* and *proADF7::gADF7; adf7 adf10* pollen tubes. These findings suggest that phosphorylation of ADF7 at Ser128 is biologically relevant.

Indeed, we obtained material from *proADF7::gADF7S128D; adf7 adf10* plants and we examined the actin cytoskeleton in both *proADF7::gADF7S128D; adf7 adf10* pollen tubes and *proADF7::gADF7S128A; adf7 adf10* pollen tubes. In the newly prepared Fig 5, we added the representative images of *proADF7::gADF7S128D; adf7 adf10* pollen tubes stained with Alexa-488 phalloidin and we also quantified the amount of F-actin in the pollen tubes.

*4. In addition, it worth to check if CDPK16-eGFP and ADF7 show some co-localizations in pollen tubes. This will provide another evidence for the functional linkage of CDPK16 and ADF7*.

Response: We agree that examination of the colocalization of CDPK16 with ADF7 in pollen tubes will provide further evidence to support the functional linkage of CDPK16 and ADF7. We therefore crossed *proCDPK16::gCDPK16-eGFP; cdpk16* with *proADF7::ADF7-mCherry;adf7* during the revision and obtained *proCDPK16::CDPK16-eGFP,proADF7::ADF7-mCherry;cdpk16adf7* plants. Unfortunately, we found that the CDPK16-eGFP signal became very dim after segregation and we failed to obtain any useful images to indicate the colocalization of CDPK16 and ADF7 in pollen tubes. However, considering that ADF7 localizes along the entire pollen tube (Zheng et al., 2013, Plant Cell. 2013, 25(9):3405-23; Jiang et al., 2022, Plant J. 2022,110(4):1068-1081) and ADF7 interacts with CDPK16 both *in vitro* and *in vivo*, it is likely that colocalization between ADF7 and CDPK16 occurs in pollen tubes.

Rev. 2:

*The current manuscript by Wang et al is the second in a developing series that describes the identification of a kinases which regulate key actin binding proteins (in this case, ADF), required for the modulation and regulation of actin cytoskeletal dynamics. In the current study, the authors demonstrate that CDPK16 interacts with ADF7, and that this interaction/activity enhances ADF7-mediated actin depolymerization and severing. Using a genetics-based approach, they observed that the rate of actin turnover is reduced in cdpk16 mutant pollen. CDPK16 phosphorylates ADF7 at Ser-128.*

*At an experimental level, the current manuscript is sound; it describes previously unknown activities of CDPK16 and ADF. At a broader, fundamental level, the current study builds upon known mechanisms and activities required for the regulation of ADF activity.*

Response: Thanks for the encouraging comments.

*Based on previous work by the Huang lab, as well as those of others, I feel it would be necessary to delve into the specificity of this interaction a bit more. Previous work from the Huang lab identified a kinase required for the regulation of ADF4 activity. Does CDPK16 p-late ADF4? I recognize that they tested ADF10, which is also expressed in pollen tubes. I believe it would be useful to evaluate the kinase interaction activity of a third ADF - one that is not expressed in pollen.*

Response: As suggested, we examined the effect of CDPK16 on the activity of ADF4 and found that CDPK16 only weakly, albeit significantly, enhances the actin depolymerizing activity of ADF4 (see the newly generated results in S5 Fig in this revised manuscript). But we lack direct evidence that CDPK16 phosphorylates ADF4, and we do not know the residues in ADF4 that might be targeted by CDPK16.

*Related to the above, the Huang group evaluated CKL2 (Plant Cell, 2016). Does CKL2 p-late ADF7? I do not mention these recommended experiments for the sake of adding additional tasks, yet to express that the authors, and others, are developing models which demonstrate a mechanism that describes specificity of ADF activity through phospho-regulation and residue specificity. It is important, that as this develops, to ensure a stringent analysis.*

Response: In addition, we also determined the effect of CKL2 on the function of ADF7. Using high-speed F-actin cosedimentation experiments, we found that CKL2 promotes the actin depolymerizing activity of ADF7, which suggests that CKL2 interacts directly with ADF7. Again, we lack direct evidence regarding whether CKL2 regulates ADF7 by phosphorylation, and we do not know which residue is targeted by CKL2. We believe that it is worth performing a deep dissection to understand how the different kinases coordinate with the different ADF isovariants. Such an investigation deserves its own independent story and is beyond the scope of the current investigation.

*What is the biological consequence of, for example, a cdpk6 mutant, or a adf7/Ser-128 plant? Are there reproductive consequences of these isoforms? I fully recognize that the current study describes an impact on the individual filament dynamics, but what is the "next step"? What is the impact on the biology of the cell, the tissue, or the plant on these modifications?*

Response: The goal of this manuscript is to document the regulation of actin dynamics in pollen tubes. We therefore focused on characterizing the function of the CDPK16-ADF7 interaction in the regulation of actin dynamics in pollen tubes. The direct consequence of altering this interaction is that the rate of pollen tube growth is reduced. Thus, we observed reduced pollen tube growth in *cdpk16* mutant pollen tubes and in pollen tubes harboring phospho-mimic ADF7 (S128D) or non-phosphorylatable ADF7 (S128A). To date, we do not know whether disruption of this mechanism has other consequences at the cell, tissue and organism level. Of course, this will be worth examining in the future.

Rev. 3:

*In their manuscript, Wang et al. propose a change in the current regulatory paradigm of ADF factors, regarding their actin depolymerizing and severing activity. The authors propose that ADF proteins (particularly ADF7) get phosphorylated in position S128, activating their depolymerizing and severing function. The authors identify a CDPK protein (CDPK16) to be involved in this regulatory process.*

*The paper is well written, and the authors combine in vivo and in vitro approaches to shed light on this regulatory process. However there would be interesting questions to address:*

Response: Thanks for the encouraging comments.

Major:

*- The authors describe that cdpk16 knock-out mutant has a reduced rate of acting turnover, by accumulating actin filaments. Next, The authors connect CDPK16 to ADF7 in vivo and in vitro, by Luciferase interaction assays and through enrichment of CDPK16 hits when pulling down ADF7. Suggesting CDPK16 to be an important regulator of the phosphorylated status of ADF7. However, the authors seem to be able to overcome the cdpk16 phenotype by overexpressing ADF7. If ADF7 needs to be phosphorylated to activate is depolymerization function, How do the authors explain this data? Are there other cdpks in the tissue that could replace cdpk16?*

Response: Non-phosphorylated ADF7 retains reasonable actin depolymerizing activity, whereas phosphorylation of its Ser128 by CDPK16 upregulates its actin depolymerizing activity. In *cdpk16* mutant pollen, although the total amount of ADF7 protein does not change compared to WT pollen, the total actin-depolymerizing activity mediated by ADF7 is reduced, which causes defects in actin turnover in *cdpk16* pollen. After we introduce more ADF7 protein into *cdpk16* pollen through overexpression, the total actin-depolymerizing activity mediated by ADF7 is increased. Overexpression of exogenous ADF can compensate to some extent for the reduction in ADF-mediated actin-depolymerizing activity resulting from the deficiency in CDPK16-mediated phosphorylation.

We noticed that other pollen-expressed CDPKs can phosphorylate ADF7 *in vitro*. However, we do not know whether they can replace the role of CDPK16, as they exhibit different subcellular localizations in pollen. In our lab, we are on the way to dissecting the physiological significance of regulation of ADF7 by other CDPKs in pollen.

*- To see the in vivo relevance of CDPK16 in phosphorylating ADF7 in the Ser128 residue, the authors should check how much of ADF7 S128 phosphorylated they find with their specific ADF7-Ser antibody in a cdpk16 mutant vs wt. Is the pool of non-phosphorylated vs phosphorylated protein substantially different? Alternatively, if the antibody would not be sensitive enough they could do an MS comparison between ADF7 in the cdpl16 vs Wild-type background.*

Response: We totally agree with this reviewer that comparison of the amount of phosphorylated ADF7 between *cdpk16* and WT using western-blot analysis probed with anti-phospho-ADF7(Ser128) antibody or by MS analysis is important in order to determine the *in vivo* relevance of CDPK16 in phosphorylating ADF7 in the Ser128 residue. Toward this end, we put efforts on generating tagged version of ADF7 in the background of *cdpk16* via crossing *cdpk16* with *proADF7::8His-gADF7; adf7*, as anti-phospho-ADF7(Ser128) antibody fails to detect phosphorylated ADF7 from total pollen extract but can recognize pulled down phosphorylated ADF7. Unfortunately,we lost the transgenic materials at T2 generation due to the influence by Covid-19, as the students cannot enter the campus. In terms of comparison by MS analysis, we met difficulties as well, as we could only detect a little of p-S128 in ADF7 even in the WT pollen, it is therefore notorious difficult to compare the difference in the amount of phosphorylated ADF7 at its Ser128 between *cdpk16* and WT.

We are very frustrated that we could not obtain direct evidence to compare the difference between *cdpk16* and WT in terms of the amount of phosphorylated ADF7 at its Ser128. However, we are quite confident that CDPK16 can phosphorylate ADF7 at its Ser128 in pollen. First of all, we have strong evidence that CDPK16 can phosphorylate ADF7, which was detected by two-dimensional electrophoresis approach showing that the amount of phosphorylated ADF7 is reduced in *cdpk16* pollen (Fig 2G, left panel). During the revision, we also determined the amount of phosphorylated ADF7 in pollen from *CDPK16* overexpressors by the same approach and found that the amount of phosphorylated ADF7 increased compared to WT, see the newly prepared data in the right panel of Fig 2G. These data together strongly suggests that CDPK16 can phosphorylate ADF7 in pollen. Considering that Ser128 in ADF7 is the major site targeted by CDPK16 *in vitro* (Fig 4C) and the effect of *CDPK16* overexpression is suppressed in pollen harboring ADF7^S128A^ (12 Fig), we are quite confident that CDPK16 regulates the function of ADF7 mainly by phosphorylating its Ser128. We hope that these data together can also convince this reviewer that CDPK16 regulates the function of ADF7 mainly by phosphorylating its Ser128.

*- The authors try to express a phosphomimic version of ADF7, S128D in planta to analyze its role in actin turnover. The mutant does not behave as an activator but rather the contrary as expected, which, as the authors suggests, could be due to the fact that the change to Aspartate would not fully mimic the function of a phosphorylated residue. To prove the role active role of ADFS128 phosphorylated, the authors could overexpress CDPK16 with a strong pollen specific promoter (pACA9) into their backgrounds pADF7:ADF7S128A and pADF7:ADF7 the adf7adf10 background. In this case they should increase the pool of phosphorylated ADF7S128 and see its enhanced effect in actin depolymerization.*

Response: We totally agree with this reviewer that ADF7^S128D^ likely does not fully mimic the function of a phosphorylated residue. As suggested, we generated *CDPK16* overexpressors in the backgrounds of pADF7:ADF7S128A; *adf7adf10* and pADF7:ADF7;*adf7adf10*, and we determined the pollen germination rate in the presence of LatB. We found that the LatB-sensitive pollen germination phenotype associated with *CDPK16* gain-of-function is suppressed in pollen harboring pADF7:ADF7S128A in *adf7adf10*. See the newly prepared data in S12 Fig in this revised manuscript.

*- The authors should also include gel filtration profiles of the proteins they use in their in vitro analyses in the supplementary data, of wild type and mutants. To ensure the proteins are properly folded, especially in the case of the mutants used in their different in vitro assays.*

Response: Our *in vitro* functional data showed that ADF7^S128A^ retains roughly similar actin depolymerizing activity to WT ADF7, and ADF7^S128D^ functions slightly better than WT ADF7 (Fig 4F-4J). These results suggest that the mutant ADF7 proteins are very likely folded properly.

Minor:

*In figure S8 the authors forgot to include marker in the figures C and D. Also in figure C is a bit confusing to know which lanes contain ADF7, maybe that could be indicated.*

Response: We have fixed these problems in this revised manuscript.

__________________________________________________

In compliance with data protection regulations, you may request that we remove your personal registration details at any time. (Use the following URL: [https://www.editorialmanager.com/pbiology/login.asp?a=r).](https://www.editorialmanager.com/pbiology/login.asp?a=r).) Please contact the publication office if you have any questions.
