# Supplementary material for: Activation of actin-depolymerizing factor by CDPK16-mediated phosphorylation promotes actin turnover in Arabidopsis pollen tubes
Source: PLoS Biol. 2023 Apr 3;21(4):e3002073. doi: 10.1371/journal.pbio.3002073 (PMC10101649; doi:10.1371/journal.pbio.3002073)
Supplement: Attachment — Submitted filename: Response to Reviewers comments.docx [file pbio.3002073.s044.docx]

**Response to Reviewers’ Comments**

We thank the three anonymous reviewers for the encouraging comments. In this revised manuscript, we have tried our best to fully address the raised concerns. See the detailed response below.

Rev. 1:

The authors have answered most of the questions I raised, and the new data they added in the revised manuscript provide more evidence to sustain their finding. But, still I have another two minor questions for the authors to address:

1. The title of the manuscript is too brief, which did not include the main findings. The specific function and biological significance of CDPK16-mediated phosphorylation of ADF7 is suggested to be highlighted in the title and in abstract.

Response: We changed the title to “Activation of actin-depolymerizing factor by CDPK16-mediated phosphorylation promotes actin turnover in *Arabidopsis* pollen tubes” as suggested.

2. The main point of the manuscript is that CDPK16-mediated phosphorylation upregulates ADF7 and most of the data supports this argument. However, it's somewhat inconsistent when it comes to the phosphorylation site S128 of ADF7. "ADF7S128D has reduced capability in promoting actin turnover in pollen compared to ADF7". This is a confusing part of the manuscript. The authors simply explain that ADF7S128D cannot fully mimic the function of phosphorylated ADF7 in pollen. Could the author give more specific explanation or speculation? The possible reasons need to be carefully analyzed and added in the discussion section. Is it possible that the other phosphorylation sites of ADF7 is involved?

Response: Thank you for pointing this out. We discussed a little bit more about the potential reason about why ADF7^S128D^ cannot fully mimic the function of phosphorylated ADF7, please find the highlighted additions.

Rev. 2:

In the second revision of this manuscript, I would like to acknowledge that the authors have addressed not only my primary concerns (in both rounds of review), but have addressed the major (and minor) elements noted by the 2 additional reviewers. As noted in my previous reviews, this is a sound manuscript and builds upon current developing work in the field of actin biology. I have no additional comments, and I believe that this manuscript will be a substantial contribution to the field.

Response: Thanks Reviewer 2 for the encouragement.

Rev. 3:

The authors have addressed the majority of the comments improving the manuscript.

Response: Thanks Reviewer 3 for the encouragement.

__________________________________________________
